# Integrating Cu_2_O Colloidal Mie Resonators in Structurally Colored Butterfly Wings for Bio-Nanohybrid Photonic Applications

**DOI:** 10.3390/ma17184575

**Published:** 2024-09-18

**Authors:** Gábor Piszter, Krisztián Kertész, Dávid Kovács, Dániel Zámbó, Ana Cadena, Katalin Kamarás, László Péter Biró

**Affiliations:** 1 Institute for Technical Physics and Materials Science, HUN-REN Centre for Energy Research, Konkoly Thege Miklos út 29-33, H-1121 Budapest, Hungary; 2 Institute for Solid State Physics and Optics, HUN-REN Wigner Research Centre for Physics, Konkoly Thege Miklos út 29-33, H-1121 Budapest, Hungary; 3 Department of Chemical and Environmental Process Engineering, Faculty of Chemical Technology and Biotechnology, Budapest University of Technology and Economics, Műegyetem rkp. 3, H-1111 Budapest, Hungary

**Keywords:** bio-nanohybrid, photonic nanostructure, butterfly wing, epicuticular wax, Cu_2_O nanoparticles, UV–visible spectroscopy, *n*-alkane

## Abstract

Colloidal Cu_2_O nanoparticles can exhibit both photocatalytic activity under visible light illumination and resonant Mie scattering, but, for their practical application, they have to be immobilized on a substrate. Butterfly wings, with complex hierarchical photonic nanoarchitectures, constitute a promising substrate for the immobilization of nanoparticles and for the tuning of their optical properties. The native wax layer covering the wing scales of *Polyommatus icarus* butterflies was removed by simple ethanol pretreatment prior to the deposition of Cu_2_O nanoparticles, which allowed reproducible deposition on the dorsal blue wing scale nanoarchitectures via drop casting. The samples were investigated by optical and electron microscopy, attenuated total reflectance infrared spectroscopy, UV–visible spectrophotometry, microspectrophotometry, and hyperspectral spectrophotometry. It was found that the Cu_2_O nanoparticles integrated well into the photonic nanoarchitecture of the *P. icarus* wing scales, they exhibited Mie resonance on the glass slides, and the spectral signature of this resonance was absent on Si(100). A novel bio-nanohybrid photonic nanoarchitecture was produced in which the spectral properties of the butterfly wings were tuned by the Cu_2_O nanoparticles and their backscattering due to the Mie resonance was suppressed despite the low refractive index of the chitinous substrate.

## 1. Introduction

The use of the abundantly available solar radiation to fulfill various needs of human civilization is gaining increasing attention. Photovoltaic power generation [1,2,3,4], plasmonics [4,5,6,7,8,9], metasurfaces [10,11], and photocatalysis [12,13,14,15] are just a few intensively investigated methods that may help in solving some of the serious environmental challenges facing humanity. The common point in these processes is the transfer of energy between electromagnetic radiation and matter. This transfer may be based on chemical or physical processes or their combination, as in the case of heterogenous photocatalysis, where the energy of light is converted directly into the desired chemical modifications by photogenerated charge carriers and/or locally enhanced electromagnetic fields on the surfaces of photonic crystals or plasmonic nanoparticles [16]. The efficiency of the photocatalytic process can be increased by exploiting the slow light effects taking place at the surfaces of photonic crystal-type nanoarchitectures [17,18]. The energy transfer between light and the photocatalytic surface site can be enhanced by the incorporation of plasmonic nanoparticles in photonic nanoarchitectures [16] or by applying a thin, conformal semiconductor coating of ZnO, TiO_2_, etc., onto the surface of the photonic nanoarchitecture, the latter of which do not inherently possess photocatalytic properties [19,20]. In a recent paper, we reported the enhancement of the photocatalytic activity of ZnO-coated butterfly wings when Cu_2_O nanoparticles were deposited on conformally coated wings [21]. On the other hand, it was found that the deposition of the Cu_2_O nanoparticles on uncoated wings did not improve, but, on the contrary, slightly decreased the photocatalytic activity. The more detailed investigation of the interaction of the biologic photonic nanoarchitecture and the Cu_2_O nanoparticles may bring useful information in understanding this.

Cu_2_O semiconductor nanoparticles are interesting because they have photocatalytic activity [22], display size-dependent optical properties [23], act as colloidal Mie resonators and generate a physical color even in the absence of ordering [24], and may exhibit high Rydberg states, associated with extreme sensitivity to the local environment [25]. Cu_2_O is an attractive material for large-scale solar energy conversion at a low cost, due to the abundant nature of copper and oxygen and the suitable bandgap for the absorption of visible light [26], as well as the effective, less energy-intensive synthesis processes [27]. The integration of such nanoparticles into photonic nanoarchitectures may present multiple routes to enhance the efficiency of the light–matter interaction. The high-index resonant dielectric nanostructures form building blocks for novel photonic nanoarchitectures with low losses and advanced functionalities [28]. Cu_2_O nanoparticles can be synthesized with a controlled shape and size in an aqueous environment [29] and can be stored for a long time when dispersed in ethanol [23]. For practical applications, Cu_2_O nanoparticles have to be immobilized on a certain substrate. Butterfly wings, with a structural color and intricate surface structures from the nanometer to centimeter scale [30,31,32,33], are particularly well suited for this purpose [21,34]. In our earlier work, we successfully confirmed that the Cu_2_O nanoparticles—after being deposited on butterfly wings—could withstand 2 h of stirring without being washed away during photodegradation measurements [21]. The structural color of butterflies, which is used most frequently in sexual communication, has remarkable stability both in time and over large geographical distances [35,36,37]. Therefore, by breeding butterflies under controlled conditions [37], one may obtain large numbers of photonic crystal-type samples with uniform structural and optical properties in a cheap and environmentally friendly way. This is a similar approach to the traditional production technology of natural silk by domesticated *Bombyx mori* moths [38,39]. Nowadays, this technology produces several thousands of tons of natural silk; its worldwide production in 2021 amounted to 86,311 tons [40].

Butterfly wings are superhydrophobic [41,42,43,44]. This property arises, on one hand, from the micro- and nanoscale morphology of the wing scales and from the native wax layer covering the insect exocuticle, constituted mainly by *n*-alkanes [45,46,47,48]. The solid *n*-alkanes with carbon atom numbers from 20 to 44 are soluble in ethanol at room temperature [49]; moreover, their solubility has a strong temperature dependence [49,50]. Therefore, the presence of the wax in butterfly wing scales may influence the outcomes of nanoparticle deposition from ethanol-based solutions. Understanding the temperature dependence of the wax removal process helps in achieving more reliable sample preparation.

This paper focuses on the preparation of 3D hybrid nanoarchitectures consisting of a bio-based template and Cu_2_O nanoparticles. We investigated the dissolution of the wax present in the wing scales of male *Polyommatus icarus* butterflies and the possibility of the removal of the wax prior to the deposition of the Cu_2_O nanoparticles, and we compared the optical properties of the nanoparticles deposited on butterfly wings, glass slides, and Si(100) single crystals. The glass and Si substrates were used as a comparison to the butterfly wing (n_chitin_ = 1.56), using one insulator with a low refractive index (n_glass_ = 1.5) and a semiconductor with a high refractive index (n_Si_ = 3.4).

## 2. Materials and Methods

### 2.1. Materials

The blue wings of Common Blue male butterflies, *Polyommatus icarus* (Rottemburg, 1775) (Arthropoda: Insecta: Lepidoptera: Lycaenidae), were used in this study. This butterfly species is not subjected to any restrictions. The range of the species covers the entire Palearctic region [51], and, recently, their presence was reported in the Nearctic too [52]. Their structural color variation in a given local population is limited to ±10 nm [53], while a spectral difference in the order of only 20 nm can be found between specimens originating from Europe and Asia [35,36]. The structural color is also resilient to environmental influences, which facilitates the laboratory breeding of the species [37]. The samples used in this study were bred in a custom-made insectarium [37].

For the octahedral Cu_2_O nanoparticles, slight modifications and upscaling were applied in a recently published protocol [54]. Briefly, 1 mL Cu(NO_3_)_2_ solution (0.1 M) was added to 91.8 mL ultrapure water in a Schott glass and stirred for several minutes. Upon introducing the base solution (200 μL of 1 M NaOH), the solution turned light blue due to the formation of Cu(OH)_2_, which was reduced by the swift addition of a hydrazine solution (3 mL, 0.2 M) under vigorous stirring. The solution turned orange within the first minute, and the growth proceeded for 10 min. The particles were collected and washed via centrifugation and redispersion with ethanol–water mixtures (50:50 *V*/*V*%), and the Cu_2_O nanoparticles were redispersed in 105 mL of absolute ethanol. The stock solution was prepared by redispersing the washed particles in 10 mL of absolute ethanol. Then, from this solution, 796 µL was diluted to 10 mL with absolute ethanol to reach a Cu_2_O concentration of 0.045 M. This latter solution was drop-cast on the wings. The Cu_2_O nano-octahedra were derived from the same batch as the ones used in ref. [21] and had a base edge length of 136 ± 12 nm. The SEM, TEM and XRD characterization of the nanoparticles and their measured optical extinction spectra were reported in refs. [21,23].

### 2.2. Sample Preparation

The sample preparation followed the same general outline as used in ref. [21], with a major difference being that the original PTFE frames were replaced with glass frames and a new high-temperature wax removal procedure described below was applied. The samples were prepared after removing all four wings from the body of the dried butterfly specimen. The wings were fixed onto glass slides using a very thin layer of poly(methyl methacrylate) (PMMA) and were left to dry overnight. Their reflectance spectra were measured the next day using an integrating sphere setup. After the measurement, two different treatments were applied with absolute ethanol (≥99.8%, AnalaR Normapur, VWR Chemicals, Radnor, PA, USA) to remove the epicuticular waxes from the surface of the photonic nanoarchitecture: overnight soaking (~16 h) at room temperature (ETA) or 8 h soaking at 50 °C (ETA50).

The Cu_2_O sol was used to prepare samples on clean butterfly wings by drop casting. PDMS rings (silicone sealing ring for GL threads (16 × 8 mm), DWK Life Sciences, Mainz, Germany) were pressed mechanically onto the surface of the glass-mounted wing to avoid the leakage of the sol, similar to the procedure used in ref. [55]. We used 120 µL of the sol introduced in the 8-mm-diameter central opening of the ring, measured with a Biohit Proline (Biohit Healthcare Ltd., Helsinki, Finland) automatic pipette. The samples were left to dry overnight; then, the PDMS rings were removed, and the samples were characterized. Glass and Si samples were prepared using the above-mentioned protocol.

### 2.3. Microscopy

The presented optical micrographs and images with an extended depth of focus (EDF) were obtained using a Zeiss Axio Imager A1 (Carl Zeiss AG, Jena, Germany) and a Nikon Eclipse LV150N (Nikon Instruments, Tokyo, Japan), respectively.

For scanning electron microscope inspection, wing pieces of a few mm^2^ were cut and fixed on metallic sample holder stubs with conductive tape. In order to maintain the original state of the samples, no other treatment was applied. Images were taken using a Scios 2 DualBeam (Thermo Fisher Scientific, Waltham, MA, USA) device.

Cross-sections of the wings used for TEM examination were prepared first by embedding the pieces of the wings in a specific resin (EMbed 812, Electron Microscopy Sciences, Hatfield, PA, USA). These samples were then cut into 70-nm-thick sections with an ultramicrotome and transferred to copper grids. The sections were examined using a TECNAI 10 (FEI Company, Hillsboro, OR, USA) transmission electron microscope.

### 2.4. Spectroscopy

Attenuated total reflectance spectra were taken by a Bruker Vertex 80 (Bruker Corporation, Billerica, MA, USA) Fourier-transform spectrometer using a multibounce (45 reflections) KRS5 ATR crystal. The solutions were drop-cast on the surface of the ATR crystal and allowed to dry at room temperature. For each measurement, 128 scans with a 4 cm^−1^ spectral resolution were taken. The resulting ATR intensities were converted to absorbance and baseline-corrected.

Reflectance spectroscopy measurements were conducted using an Avantes (Avantes BV, Apeldoorn, The Netherlands) modular fiber-optic system consisting of a high-sensitivity spectrophotometer (AvaSpec-HSC1024x58TEC-EVO), a stabilized deuterium–halogen light source (AvaLight-DH-S-BAL), an integrating sphere (AvaSphere-30-REFL), and a white diffuse tile (WS-2) as a reference. Microspectrophotometry was conducted using the above-mentioned setup supplemented with a custom-made adapter tube, which allowed the attachment of the spectrophotometer to the 100× objective (LD C Epiplan-Neofluar 100×/0.75, FWD = 4.0 mm, Carl Zeiss AG, Jena, Germany) of a Zeiss Axio Imager A1 optical microscope.

For hyperspectral imaging, we used a custom-made setup consisting of an Optics Focus (Beijing, China) Motorized XY-Axis Stage and Avantes normal-incidence bifurcated fiber-optic probe (FCR-7UV200-ME-SR). A custom LabView (Austin, TX, USA) application was used to synchronize the movement of the stage and for the storage of the data from the spectrometer. A detailed description of the setup can be found in ref. [56]. Due to the angular dependence of the reflectance on the local incident angle when using the normal-incidence probe of the fiber-optic spectrometer to carry out the hyperspectral measurements, the measured reflectance values exhibited a larger standard deviation as compared to integrating sphere measurements.

## 3. Results

### 3.1. Structure of Dorsal Wing Surface and Cover Scales of Male P. icarus Butterflies

**Figure 1:**
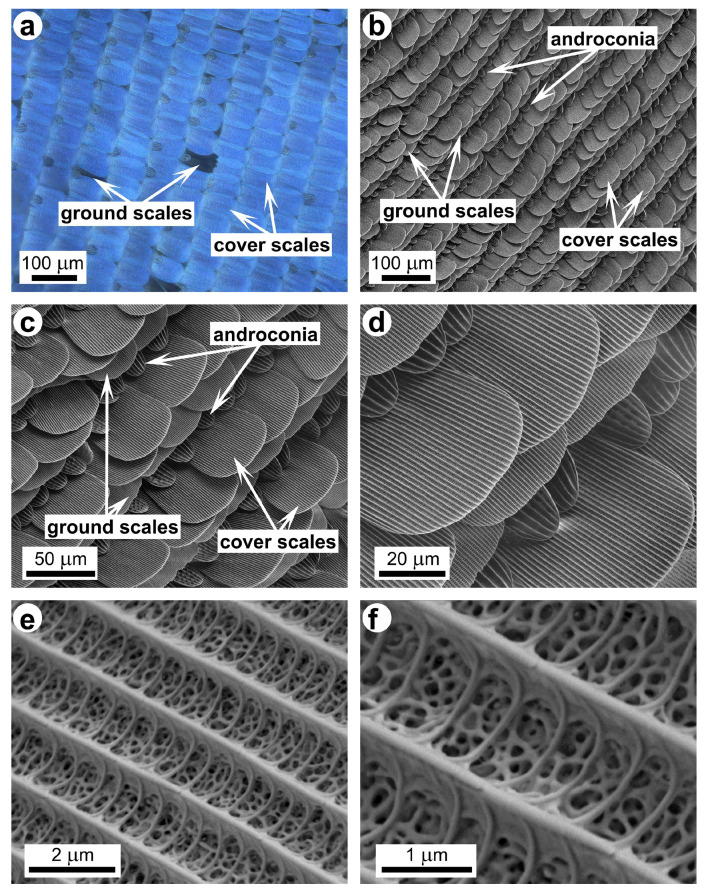
Photonic nanoarchitecture in the blue dorsal wing scales of a male *Polyommatus icarus* butterfly. (**a**) Optical microscope image of the wing scales; (**b**–**f**) SEM images showing the details of the photonic nanoarchitecture in increasing magnification steps; (**b**) overview of the scales on the dorsal wing surface; (**c**) detailed view of the three types of scales on the dorsal wing surface; (**d**) the blue color generating cover scales; (**e**) the blue color generating photonic nanoarchitecture; (**f**) a high-magnification detail of the blue color generating photonic nanoarchitecture.

**Figure 2:**
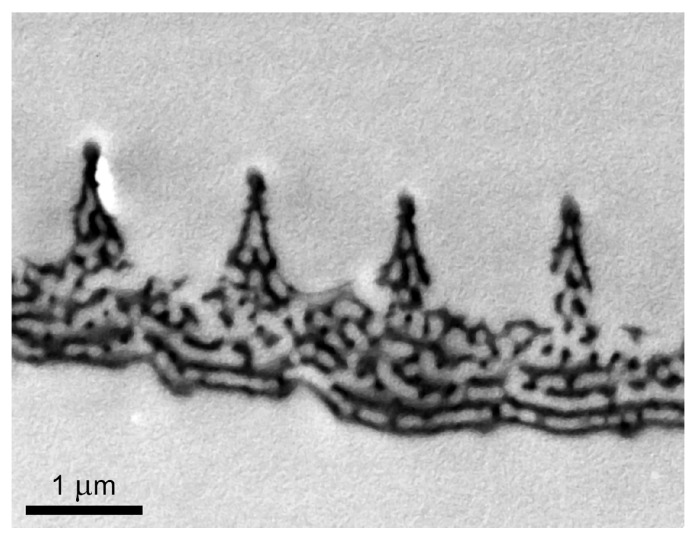
Cross-sectional transmission electron microscope image of a male *P. icarus* cover scale. The photonic nanoarchitecture with a complex 3D structure can be seen in the lumen of the scale.

The dorsal wing surface color of the male *P. icarus* butterfly—as in the case of many other Lycaenid males—is dominantly and uniformly blue–violet [53]. When examined with an optical microscope, one may observe three types of scales (Figure 1a): (i) the upper layer of blue-colored cover scales; (ii) the dark brown ground scales below them; and (iii) the androconia, which are better seen in the low-magnification scanning electron microscopy image (Figure 1b–d). Gradually increasing the SEM magnification (Figure 1b–f) reveals the details of the photonic nanoarchitecture. Cross-sectional transmission electron microscopy through these scales shows that the photonic nanoarchitecture has a complex 3D structure (Figure 2), responsible for the blue color: the proper spacing of the chitinous layers of the nanoporous multilayer structure reflects light primarily in the 300–500 nm wavelength range [53].

### 3.2. Wax Dissolution in Ethanol and Acetone

**Figure 3:**
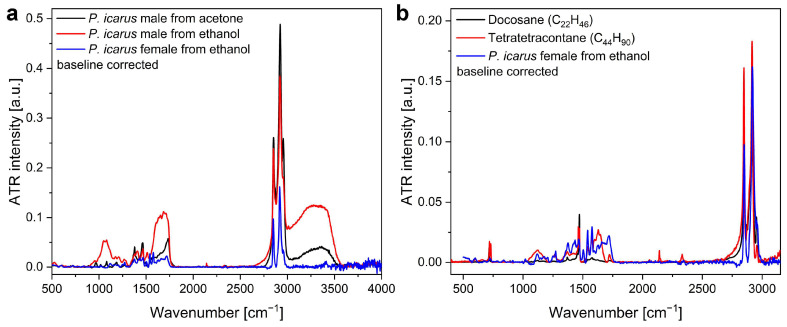
Baseline-corrected ATR spectra of *n*-alkanes and wax from *P. icarus* butterfly wings after extraction and subsequent evaporation of solvent: (**a**) male wings from ethanol and from acetone and female wings from ethanol; (**b**) *n*-alkanes vs. female wings from ethanol.

The four wings of three male *P. icarus* butterflies were detached from the bodies of the insects and soaked in acetone for 5 days at room temperature. A similar procedure was applied using ethanol to another three males and three females to determine any possible chemical composition differences between the sexes. The ethanol and acetone solutions were dried, and the residues were investigated by attenuated total reflectance infrared (ATR-IR) spectroscopy. The spectra in Figure 3, compared to the spectra of *n*-alkanes C_22_H_46_ (docosane) and C_44_H_90_ (tetratetracontane), clearly show that indeed the wings are covered by a thin wax layer constituted of such *n*-alkanes.

### 3.3. Optical Effect of Wax Removal

**Figure 4:**
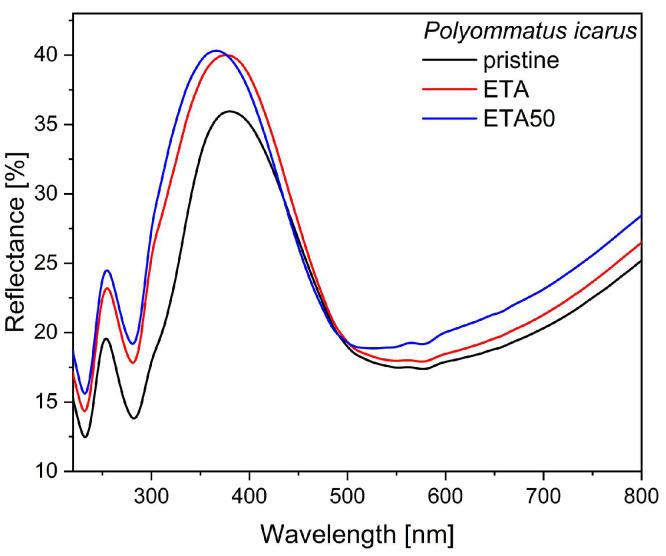
Averaged reflectance measurements with the integrating sphere on 40 wings of male *P. icarus* males: in pristine state, after ETA treatment, and after ETA50 treatment.

As the optical properties of photonic nanoarchitectures are very sensitive to the addition or subtraction of constituents, we investigated the effect of wax removal on the reflectance of the blue butterfly wings. Two different ethanol-based wax removal procedures were tested: overnight soaking (~16 h) at room temperature (ETA) and 8 h soaking at 50 °C (ETA50). Figure 4 shows the reflectance modification averaged over 40 wings (10 butterflies × 4 wings). The individual spectra and their statistical evaluations are given in Figure S1. The effect of the removal of the wax layer can be clearly identified, while the nature of the effect is dependent on the temperature at which the ethanol treatment of the wings is carried out (room temperature or 50 °C).

### 3.4. Hyperspectral Characterization

**Figure 5:**
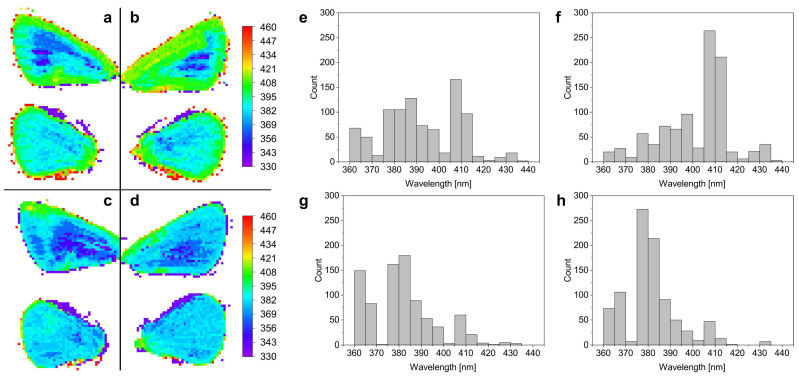
Hyperspectral reflectance characterization of the wings of a male *P. icarus* butterfly after different ethanol treatments. False color images of reflectance peak wavelengths of (**a**,**b**) wings before ETA or ETA50 treatment. False color images of reflectance peak wavelengths of wings after (**c**) ETA or (**d**) ETA50 treatment. The color scale on the right-hand side applies for all panels of the image. Histograms of reflectance peak wavelength measured on (**e**,**f**) wings before and wings after (**g**) ETA or (**h**) ETA50 treatment were calculated.

Hyperspectral imaging was carried out on the four wings of a male *P. icarus* individual before and after the ETA or ETA50 treatment. Peak wavelength maps were generated from the reflectance measured at each point, and peak wavelength histograms were calculated from these, as shown in Figure 5. In agreement with the spectral measurements carried out using the integrating sphere, the ETA50 treatment (Figure 5d) was more effective for the removal of the wax from the wings compared to the room-temperature ethanol treatment (Figure 5c), resulting in homogeneous wing surfaces with less variability in structural color.

### 3.5. Ethanol Dissolution of an n-Alkane Mixture

The same *n*-alkanes, C_22_H_46_ and C_44_H_90_—as used for the ATR-IR measurements—were mixed in equal amounts, molten, and drop-cast onto glass slides. After solidification, the samples were subjected to the ETA or ETA50 treatment, followed by optical microscopic examination (Figure S2). The microscope images clearly showed that the ETA50 treatment removed significantly more of the wax mixture as compared to the ETA treatment.

### 3.6. Cu_2_O Deposition on Butterfly Wings

**Figure 6:**
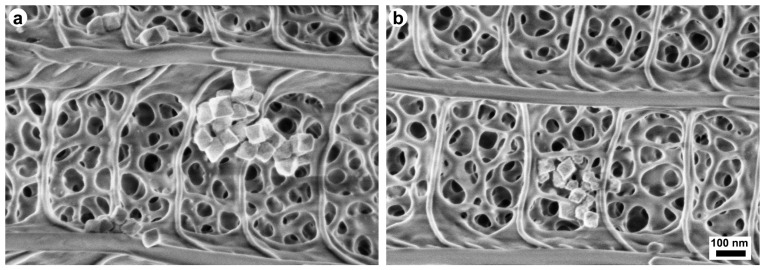
SEM images of the cover scales of a *P. icarus* wing after drop casting Cu_2_O nanoparticle sol. The deposited Cu_2_O nanoparticles can be seen (**a**) on the surface and (**b**) inside the upper layers of the photonic nanoarchitecture.

The characterized Cu_2_O nanoparticles originated from the same batch as the ones used in ref. [21] and reported in refs. [21,23]. The drop casting of the Cu_2_O nanoparticle sol was carried out after wax removal via the ETA or ETA50 treatment from the individual butterfly wings fixed onto glass slides. The SEM images of the wing cover scales show the deposited nanoparticles, which can penetrate the topmost nanoporous layers of the photonic nanoarchitecture (Figure 6a,b).

**Figure 7:**
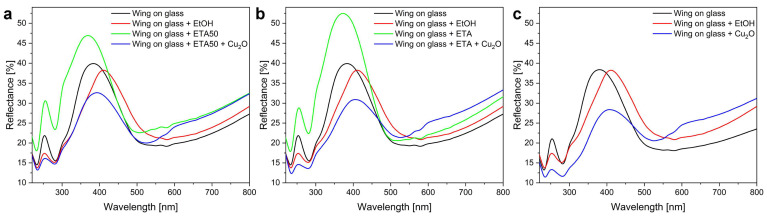
Reflectance spectra of *P. icarus* wings in glass-mounted state, after ethanol pretreatment, and after application of 120 µL of Cu_2_O nanoparticle sol. (**a**) ETA50, (**b**) ETA, and (**c**) no pretreatment wings are shown. In all panels, the red curve reflects the application of 120 µL pure ethanol on an untreated butterfly wing.

The reflectance spectra measured on the Cu_2_O nanoparticle-deposited wings after ETA or ETA50 pretreatment can be seen in Figure 7a,b. For comparison, the same amount of pure ethanol was deposited also on an untreated wing as a control sample (Figure 7c). As is shown in Figure 7, both the ETA and ETA50 treatments, similarly to the results presented in Figure 5, caused an increase in the reflectance amplitude and a shift in the peak towards UV. The subsequent deposition of the Cu_2_O nanoparticles redshifts the reflectance maximum, and this effect is dependent on the removal degree of the native wax layer. When no wax removal occurred prior to the deposition of the Cu_2_O NP sol, the structural color change was difficult to separate from the effect of pure ethanol deposition.

### 3.7. Cu_2_O Deposition on Glass and Si(100)

**Figure 8:**
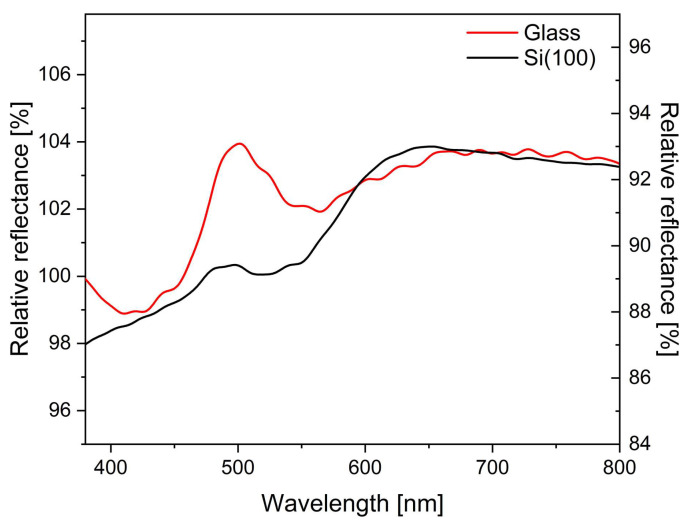
Integrating sphere reflectance spectra of Cu_2_O nanoparticles as measured with respect to the substrate. The clean glass or Si(100) surface was taken as a reference.

To test the effects of different substrates on the optical properties of the deposited Cu_2_O nanoparticles, the same sol in the same amounts as used for the butterfly wings was drop-cast on microscope glass slides and Si(100) single crystals. Reflectance measurements were carried out using the same setup and similar conditions as used for the butterfly wings, while the relative reflectance spectra were calculated using the glass/Si(100) substrate as a reference (Figure 8).

The distribution of the nanoparticles was similar on flat substrates, glass, and Si. One may observe both single or a few particles and large clusters of nanoparticles (Figure S3). The single particles have a bluish green color on glass, whereas, on Si(100), all nanoparticles have a dark orange color. These observations are confirmed by the microspectrophotometry measurements in Figure S3c,g. One has to emphasize here that, in the case of macroscopic reflectance measurement with an integrating sphere, all reflected light is collected from the upper hemisphere, while the 100× objective of the microscope used in the microspectrophotometry measurement collects the light only from a limited angular range. Despite this, the two types of measurements are qualitatively similar. The large clusters appear in an orange or dark brown color on glass (optical micrograph in Figure S3b, reflectance measured with the microspectrophotometer in Figure S3d) and on Si(100) (Figure S3f,h). Even the microspectrophotometry spectra of the large clusters on glass exhibit a small peak at around 500 nm (corresponding to the resonant Mie scattering of the nanoparticles), but this has a much smaller amplitude than the plateau over 200% in the range of 625 nm to 700 nm (corresponding to the orange color of the large aggregates).

**Figure 9:**
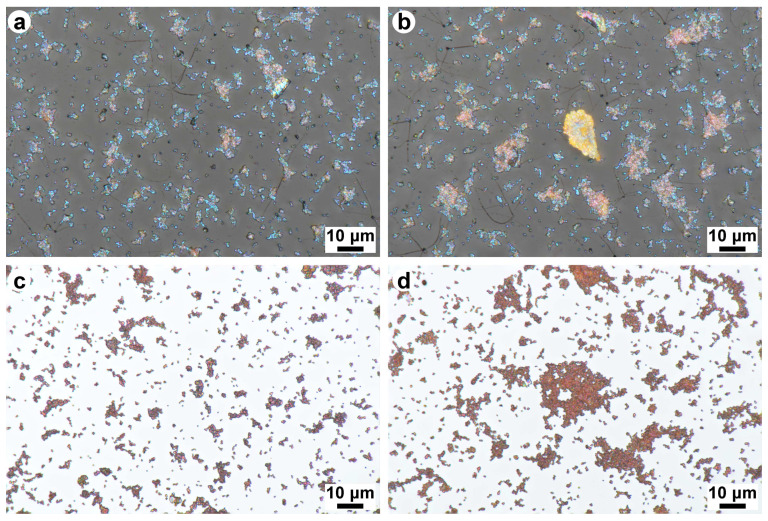
Focus stacking optical micrographs taken from Cu_2_O nanoparticles on (**a**,**b**) glass and on (**c**,**d**) Si(100). Note that, in (**a**,**c**), single particles and small clusters are visible, while, in (**b**,**d**), large clusters are also present. On glass, the small clusters clearly show a bluish green color, in good correspondence with the reflectance peak measured around 500 nm.

To test whether the observed difference between the glass and Si(100) substrates was related to the particular imaging conditions used in the microspectrophotometry setup, images were acquired using a focus stacking microscope too. The micrographs are shown in Figure 9; one may observe that the Cu_2_O nanoparticles and small clusters have a bluish green color on glass, whereas, on Si(100), they are brown–orange. The large clusters on both substrates have an orange color. The observed colors are in good agreement with the measured spectra in Figure 8 and Figure S3.

### 3.8. Optical Microscopy and Microspectrophotometry of Butterfly Wings with Deposited Nanoparticles

**Figure 10:**
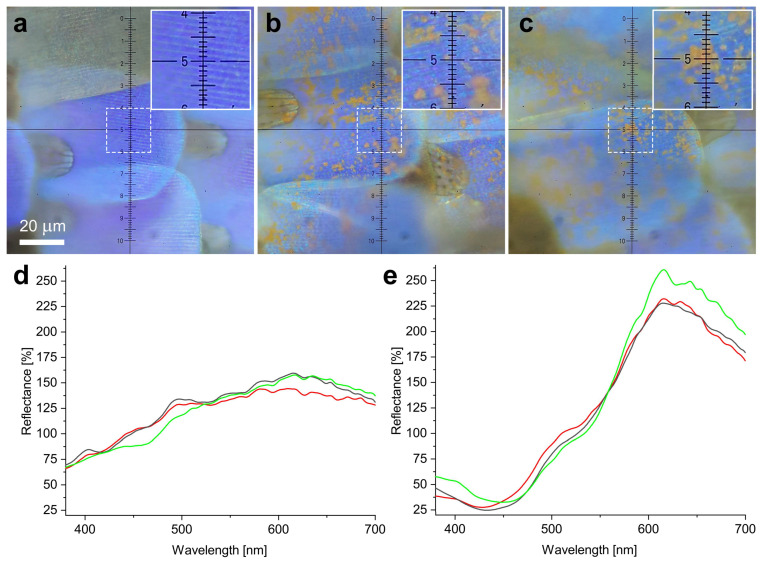
Optical micrographs and microspectroscopy reflectance measurements of Cu_2_O nanoparticles on male *P. icarus* butterfly wing scales. (**a**) Pristine butterfly wing scale that was taken as a reference for the microspectroscopy of (**d**,**e**). (**b**) Micrograph of a small cluster of Cu_2_O nanoparticles. (**c**) Micrograph of a large cluster of Cu_2_O nanoparticles. (**d**) Reflectance spectra acquired on three similar small clusters as seen in (**b**). (**e**) Those acquired on three large clusters as in (**c**).

The wing scales of an ETA50 sample with the deposited Cu_2_O nanoparticles were investigated using the same microspectrophotometry setup as used for the glass and the Si(100) samples (Figure 10). Three individual spectra are given for small particles and large clusters, in all of which the pristine wing was taken as a reference. Micrographs of pristine wing scales, of a small particle on a wing scale, and of a large Cu_2_O cluster on a scale are given. Neither the spectra taken on small particles nor those taken on large clusters show similar peaks as measured macroscopically with an integrating sphere for Cu_2_O on glass over an area corresponding to a circle with a 6 mm diameter (Figure 8) or with the microspectrophotometer for small particles (Figure S3a,b).

## 4. Discussion

### 4.1. Photonic Nanoarchitectures of P. icarus Butterflies

The photonic nanoarchitectures of male *P. icarus* butterflies are reproduced generation by generation with a very high degree of stability [35,36,37], because they play an important role in prezygotic sexual communication [53]. This makes them a very suitable platform for experiments where a large number of samples with optical properties within a narrow range of variation are needed, or even for large-scale applications if laboratory breeding is implemented [37]. We explored the conditions suitable for the integration of Cu_2_O plasmonic Mie resonators into the photonic nanoarchitectures of butterfly wings. Our work was motivated by the possibility of using such bio-hybrid nanoarchitectures in photocatalysis applied for water remediation [21]. Cu_2_O nanoparticles are intensively studied for photocatalytic applications [22,57,58]. In order to be used for water remediation applications, they have to be immobilized on a substrate, preserving their photocatalytic properties. The hierarchical structure of butterfly wings (Figure 1 and Figure 2), from tens of nanometers to tens of millimeters, may constitute a cheap and environmentally friendly solution to this problem. Beyond the advantage of the hierarchical structure, a further benefit may arise from the structural color present on the wings of many butterfly species, such as the males of *P. icarus*. The photonic nanoarchitectures of this species have many similarities to so-called photonic balls [59]. These are spheres that are tens of micrometers in diameter, containing assemblies of nanoparticles and nanopores with spacing comparable to the wavelength of light. The disordered but still correlated nanostructured building elements generate a structural color with a reduced angular dependence by a very similar mechanism to that used in the wing scales of *P. icarus* males. In model calculations [59], the high refractive index value of 1.52 and the low index value of 1 for air fit well with the case of chitin and air in butterflies (n_chitin_ = 1.56 [60,61]).

### 4.2. Wax Removal

The first modification step performed on the butterfly wings, the removal of the wax layer, has the effect that one may expect with the removal of the thin film that coats the photonic nanoarchitecture conformally: the blueshift of the main reflectance peak. The presence of the wax constituted by *n*-alkanes is clearly shown in the ATR-IR results in Figure 3. A difference in the composition of the ethanol or acetone extract of the wings was found between the males and females, which may be attributed to the presence of androconia on the wings of the males (Figure 1), responsible for the release of pheromones during courtship to attract females [62]. The androconia are absent from the wings of the females, while they have the same wax coverage as the males.

When comparing the ETA and ETA50 treatments (Figure 4 and Figure 5), one finds that the ETA50 treatment is more effective and reliable. This is a consequence of the strong temperature variation in the solubility of the *n*-alkanes in ethanol [49,50]. As the dissolution of the artificial wax layers shows in Figure S2, for the same wax layer, the ETA50 treatment was more effective in removing the wax coverage. The hyperspectral data in Figure 5 clearly support the same behavior for the butterfly wings too.

If the removal of the wax layer is not performed, then the overall effect of the application of Cu_2_O nanoparticles dispersed in ethanol would originate from two sources: the modification induced by ethanol alone and the modifications produced by the deposited Cu_2_O nanoparticles. The clear separation of the two effects may not be straightforward (Figure 6).

### 4.3. Cu_2_O Deposition on Butterfly Wings

The deposition of Cu_2_O nanoparticles decreases the amplitude of the reflectance maximum and redshifts the peak (Figure 7). Using the absorbance of the Cu_2_O nanoparticle sol from ref. [23], one can calculate the transmittance of a sparsely deposited Cu_2_O nanoparticle thin film (Figure S4a). The illuminating light has first to cross the Cu_2_O layer in order to interact with the photonic nanoarchitecture of the wing. Using the calculated transmittance of Cu_2_O NPs, the expected modification on the reflectance of the butterfly wing with the Cu_2_O nanoparticles can be estimated (Figure S4b,c) for the ETA50 wing. The differences in the measured and calculated curves in the region of the reflectance maximum of the *P. icarus* wing show that the behavior of the nanohybrid photonic nanoarchitecture is clearly different from that of a “wing + thin film filter”-type structure, as no peak shift was found at the calculated reflectance, while Cu_2_O nanoparticle deposition resulted in the redshift of the main reflectance peak. These findings are consistent with our recent results [55], where in situ grown Au nanoparticles integrated inside a photonic nanoarchitecture induced a redshift to the reflectance peak, while the physical vapor deposition of a Au thin film only showed “filter”-type behavior with the reduction of the peak amplitude.

The integration of the Cu_2_O nanoparticles in the photonic nanoarchitecture of the ethanol-pretreated wings (Figure 6) and the untreated wing was compared to the effect of the same amount of pure ethanol applied under identical conditions as in the nanoparticle sols (Figure 7). The main effect of the application of pure ethanol is the redshift of the reflectance maximum of the pristine wing, with an almost negligible reduction in the amplitude and an alteration in the shape of the reflectance maximum. These effects are attributed to the dissolution of the native wax layer and its redistribution in the deep pores of the photonic nanoarchitecture upon the evaporation of the solvent. To avoid this effect, the wax layer must be removed prior to the application of the ethanol-based sol. If this is not conducted beforehand, the modifications induced in the spectra have two sources: (i) the effect of the ethanol alone and (ii) the effect produced by the presence of the Cu_2_O nanoparticles (Figure 7).

### 4.4. Cu_2_O Deposition on Glass and Si(100)

The same amounts of nanoparticle sols as used for the butterfly wings under identical conditions were deposited on glass slides and p-type Si(100) wafers using PDMS rings with an inner diameter of 8 mm. The samples were evaluated using reflectance spectrophotometry, where clean pieces of substrates were used as references (Figure 9). While, on the glass substrate, a clear reflectance peak is found at 500 nm (Figure S3c), on the Si substrate (Figure S3g), no such peak appears. At wavelengths between 600 nm and 800 nm, both samples exhibit similar reflectance amplitude values. This range corresponds to the orange color of the bulk Cu_2_O [63,64].

Cu_2_O nanoparticles are known to be excellent Mie resonators [24]. Unlike the surface plasmon resonance of metal nanoparticles, which consists mainly of electric multipolar modes, high-refractive-index dielectric nanoparticles possess magnetic multipole modes, as well as electric ones [65]. Ohmic losses and the resultant heating in plasmonic metal nanoparticles can be avoided in dielectric Mie resonators. Another exciting feature is the possibility to tune their far-field scattering by choosing different substrates or particles of different sizes [66]. For example, the peak wavelength of a Si nanoparticle on a silica, Si, Au, or Al substrate can vary from 450 nm to 700 nm; the intensity of the scattered light can vary by a factor of ten [65]; and, depending on their size, the scattered peak wavelength of Cu_2_O nanoparticles can change from 450 nm to 650 nm [67]. In our experiment, Cu_2_O nanoparticles with a well-controlled size and octahedral shape [23] were integrated into a photonic nanoarchitecture of biological origin, producing, in this way, a bio-nanohybrid photonic nanoarchitecture with novel optical properties. The single-particle scattering of these nanoparticles, measured under dark-field epi-illumination was found to be at 480 nm on ITO [23]. When the Cu_2_O NPs were deposited on a flat glass substrate, using the same procedure as used for the butterfly wings and measuring the sample with the integrating sphere (Figure 8), a clear reflectance maximum was found at 500 nm, whereas the same NPs on p-type Si(100) do not exhibit a reflectance maximum in this range. In the region of 600 nm to 800 nm, the reflectance spectra on glass and Si are similar and show a broad plateau, which corresponds to the brown–orange color of the aggregated Cu_2_O nanoparticles. The micrographs taken with a focus stacking microscope (Figure 9) are in good agreement with the measured spectra (Figure 8). The differences found between the glass and the Si substrate show that the single Cu_2_O NPs and their “oligomers” [65] behave like Mie resonators and are characterized by the substrate modulation of their electromagnetic resonances [68,69].

Like the glass substrate, the chitin-based photonic nanoarchitecture is an insulator, and, in contrast to Si, it is a low-refractive-index material, even in bulk form (n_chitin_ was found to vary between 1.54 and 1.58 [60] or 1.6 and 1.63 [61] in the visible range). The experiments show that, on butterfly wing scales, the Cu_2_O nanoparticles do not backscatter light at around 500 nm. Considering that the effective refractive index of the butterfly photonic nanoarchitecture constituted from air and chitin (Figure 1f and Figure 2) must be lower than that of bulk chitin, the absence of the Mie scattering cannot be attributed to a simple substrate effect, as in the case of Si. On the other hand, the spectra shown in Figure 6 clearly demonstrate the absence of the reflectance peak around 500 nm, irrespective of the pretreatment applied to the butterfly wing before the deposition of the Cu_2_O NPs. The reflectance spectra taken from small particles on the butterfly wing (Figure 10d) also lack the reflectance peak at 500 nm. The spectra taken from large clusters on the butterfly wing (Figure 10e) show only the expected reflectance increase in the range of 500 nm to 700 nm, associated with the bulk color of Cu_2_O [63,64].

Such changes in Mie scattering for a Si-Au heterodimer [70] were reported earlier. The backward scattering of a Si nanoparticle with a diameter of 120 nm at 522 nm almost equals the forward scattering intensity. After the formation of the Si-Au dimer, the backward scattering at 522 nm is suppressed and a broad maximum in forward scattering appears at 600 nm. The color of Mie resonator array band-pass filters can be tuned across the visible spectrum [71]. These experimental results show that the behavior of Mie resonator nanoparticles can be altered by other nanoparticles or thin films in their vicinity [72]. The suppression of the reflectance at 500 nm measured on glass, when the Cu_2_O nanoparticles are placed on the photonic nanoarchitecture of the butterfly wing, is attributed to such effects. In contrast to the case when the Cu_2_O nanoparticles are placed on a substrate with a high refractive index, like Si, the coupling of the complex electromagnetic field of the biological photonic nanoarchitecture with the Mie-resonant particles causes the suppression of the backscattering at 500 nm and a redshift in its reflectance peak. The hybrid bio-nanoarchitecture possesses novel properties as compared to each of the constituting components. In this way, Cu_2_O nanoparticles on butterfly wings can be used to accomplish three goals at the same time: (i) to provide a suitable substrate for the immobilization of the nanoparticles, (ii) to tune the reflectance maximum of the new hybrid bio-nanoarchitecture to the desired spectral range, and (iii) to achieve the most advantageous overlap with the absorption of the pollutant to be degraded [20]. Moreover, the Cu_2_O nanoparticles themselves can increase the photocatalytic efficiency due to their photocatalytic activity in the visible range [21].

## 5. Conclusions

Butterfly wings, with their complex hierarchical nanoarchitecture, which can be produced in a cheap and environmentally friendly way, constitute a promising substrate for the immobilization of photocatalytic Cu_2_O nanoparticles and other similarly sized nano-objects. A simple ethanol pretreatment is sufficient to remove the native wax layer covering the butterfly scales. If wings possessing structural colors are used, novel bio-nanohybrid architectures of colloidal Cu_2_O nanoparticles and chitin-based photonic nanoarchitectures can be produced. We found that the Cu_2_O nanoparticles integrated well into the photonic nanoarchitecture of the *P. icarus* wing scales; they exhibited Mie resonance on the glass slides, and the spectral signature of this resonance was absent on Si(100). The novel bio-nanohybrid photonic nanoarchitectures based on butterfly wings and Cu_2_O nanoparticles exhibit properties that differ from their constituents and allow the tuning of both their spectral properties and the properties arising from the Mie resonance of the Cu_2_O nanoparticles.

## Data Availability

The original contributions presented in the study are included in the article and Supplementary Materials, further inquiries can be directed to the corresponding author.

## Supplementary Materials

The following supporting information can be downloaded at https://www.mdpi.com/article/10.3390/ma17184575/s1, Figure S1: Individual reflectance spectra and averaged spectra of 40 *Polyommatus icarus* males after different processing steps. Figure S2: Optical microscope images in reflected light of the molten mixture of C_22_H_46_ and C_44_H_90_ on glass after the ETA or ETA50 treatment. Figure S3: Optical micrographs and spectra acquired with a microspectrophotometer on Cu_2_O nanoparticles. Figure S4: Comparison of the measured reflectance spectra with the calculated ones using the absorbance spectrum of the Cu_2_O nanoparticle sol.
