# Supplementary material for: Integrating Cu2O Colloidal Mie Resonators in Structurally Colored Butterfly Wings for Bio-Nanohybrid Photonic Applications
Source: Materials (Basel). 2024 Sep 18;17(18):4575. doi: 10.3390/ma17184575 (PMC11433277; doi:10.3390/ma17184575)
Supplement: Supplementary file 1 [file materials-17-04575-s001.zip › materials-3144047-supplementary.pdf]

Supplementary Information

for

Integrating Cu_2O colloidal Mie resonators in structurally colored butterfly wings for bio-nanohybrid photonic applications

Gábor Piszter[1], Krisztián Kertész[1], Dávid Kovács[1], Dániel Zámbó[1], Ana Cadena[2,3], Katalin Kamarás[1,2], László Péter Biró[1]

1. Institute for Technical Physics and Materials Science, HUN-REN Centre for Energy Research, Konkoly Thege Miklos út 29-33, H-1121 Budapest, Hungary

2. Institute for Solid State Physics and Optics, HUN-REN Wigner Research Centre for Physics, Konkoly Thege Miklos út 29-33, H-1121 Budapest, Hungary

3. Department of Chemical and Environmental Process Engineering, Faculty of Chemical Technology and Biotechnology, Budapest University of Technology and Economics, Műegyetem rkp. 3, H-1111 Budapest, Hungary

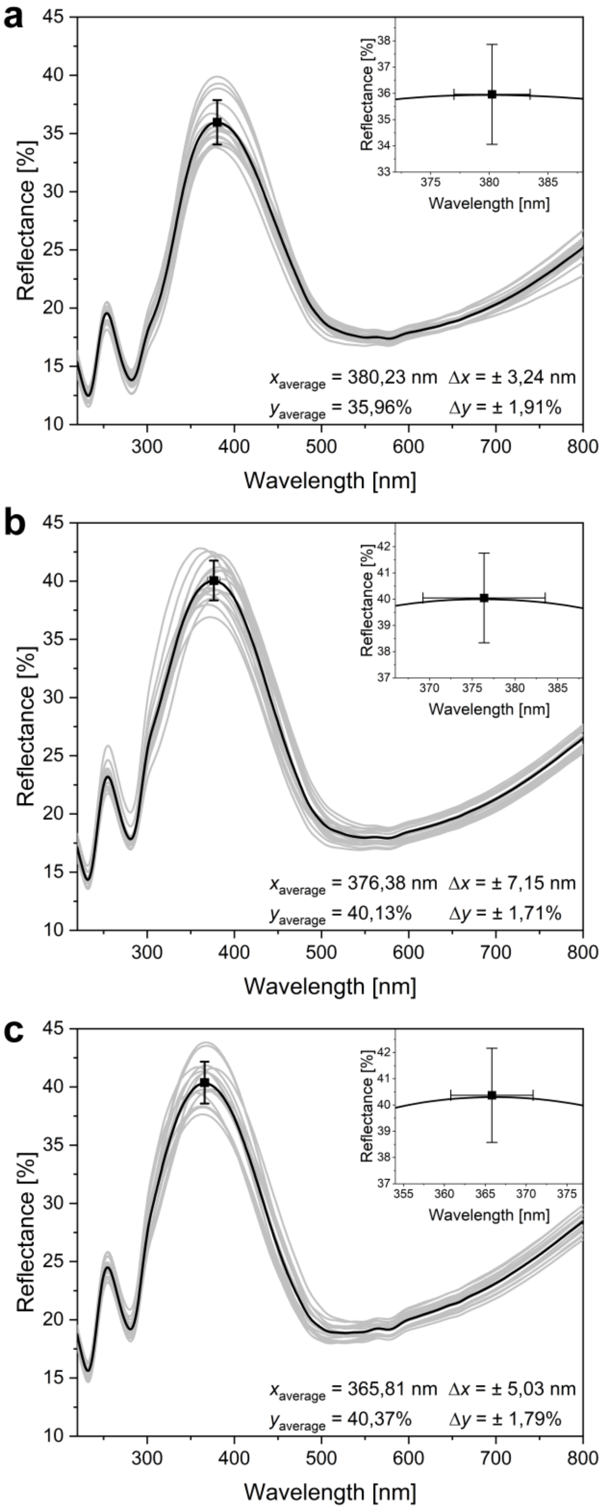

Figure S1. Individual reflectance spectra (light gray) and averaged spectra (black) of 40 *P. icarus* males after different processing steps: (a) glued onto glass substrates; (b) after ETA treatment; (c) after ETA50 treatment. Insets in the upper-right corner show the peak region and the calculated deviations, which are given numerically in the lower-right corner.

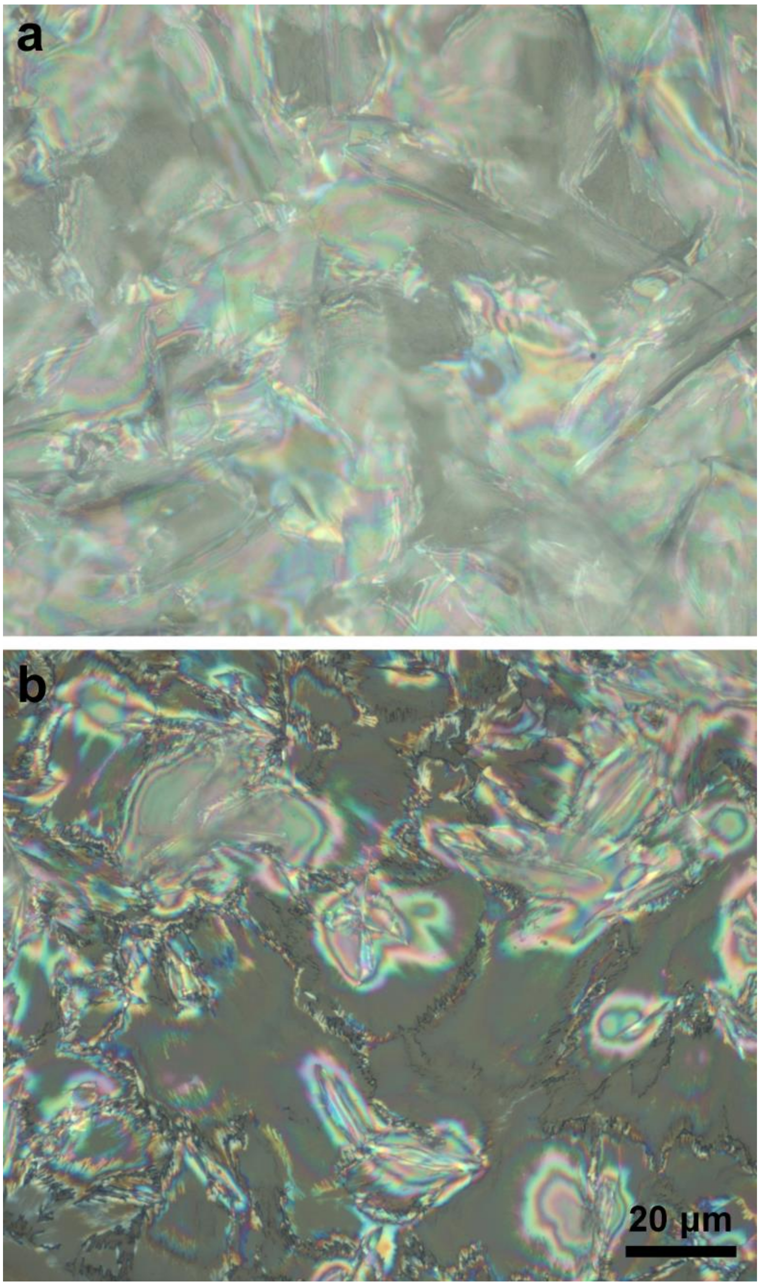

Figure S2. Optical microscope images in reflected light of molten mixture of $C_{22}H_{46}$ and $C_{44}H_{90}$ on glass after (a) ETA or (b) ETA50 treatment. The darker gray regions in (b) are the surfaces of the glass substrate without an *n*-alkane layer.

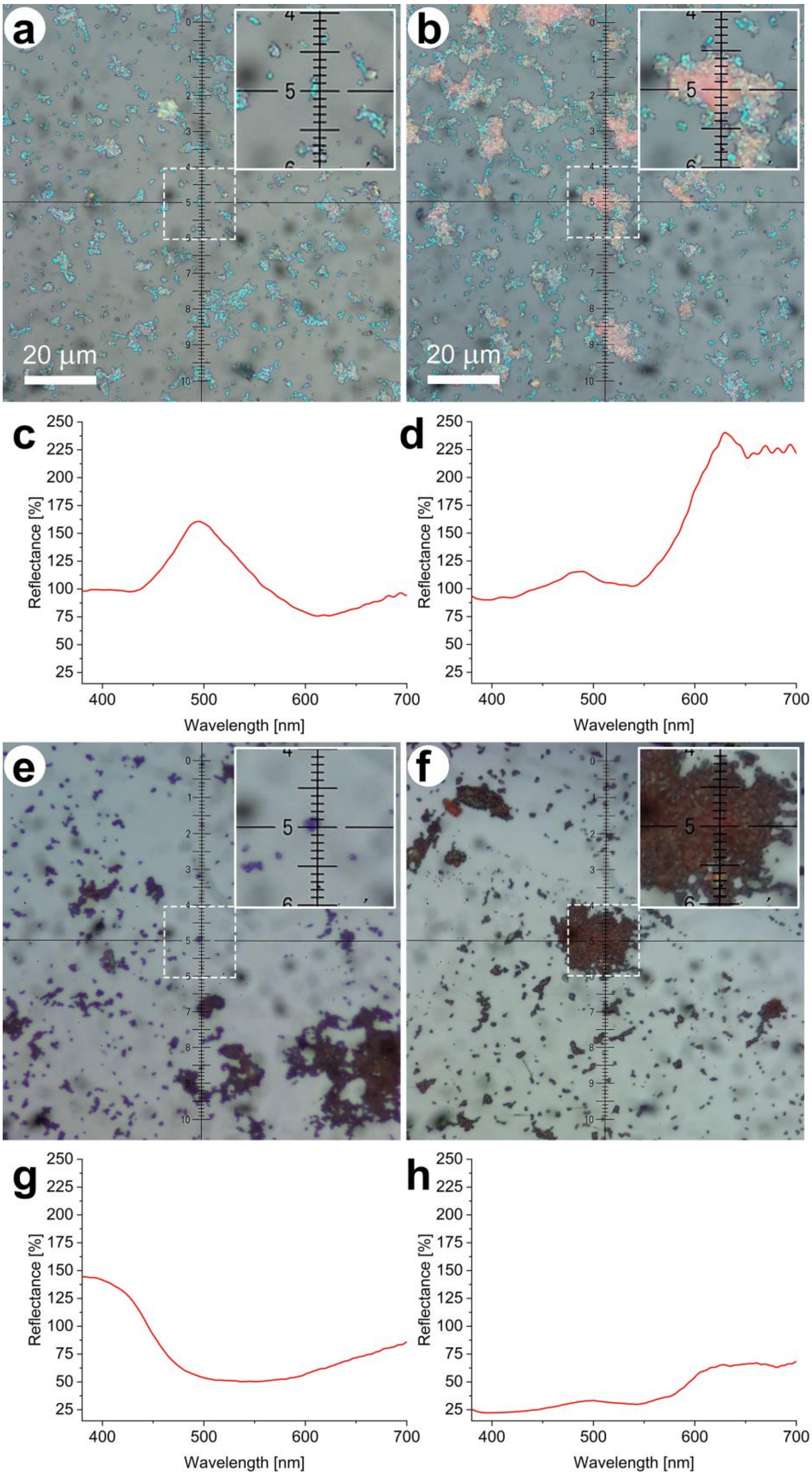

Figure S3. Optical micrographs and spectra acquired with a microspectrophotometer on (a) small or (b) large clusters of Cu_2O nanoparticles deposited on glass and (e) small or (f) large clusters deposited on Si(100). The corresponding spectra on (c, d) glass or (g, h) Si(100) are

shown below the micrographs. For all spectra, the reflectance of the clean substrate was taken as a reference.

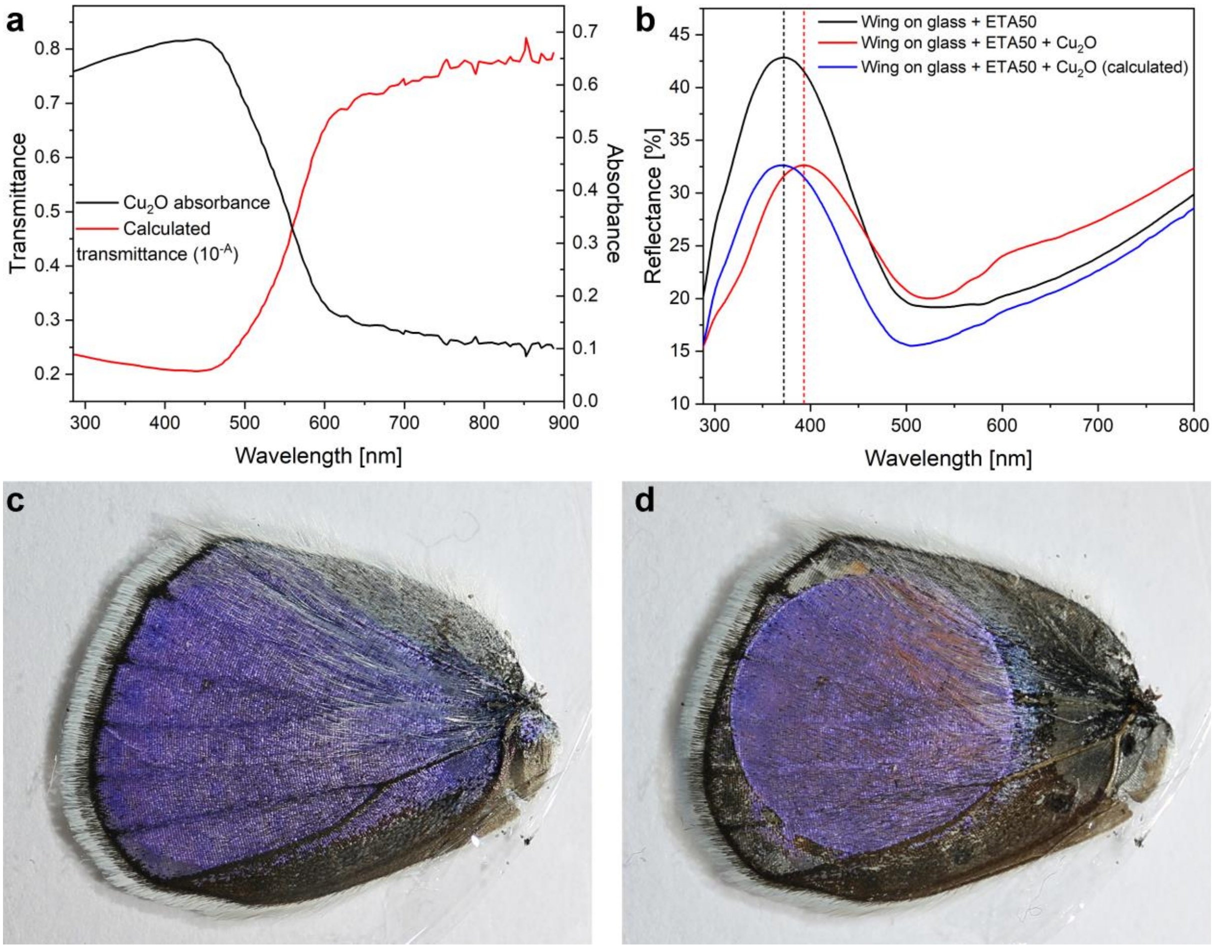

Figure S4. Comparison of the measured reflectance spectra with the calculated ones using the absorbance spectrum of Cu_2O nanoparticle sol. (a) Transmittance spectrum calculated from the absorbance given in Ref. [24]; (b) measured and calculated reflectance spectra of a wing pretreated in ethanol (ETA50) after Cu_2O nanoparticle deposition. Photographs of a wing (c) pretreated in ethanol and (d) after Cu_2O nanoparticle deposition.
